# A New Era Is Beginning in Central and Eastern Europe: Information and Communication Technology Services Exceed Manufacturing in the Global Production Chain

**DOI:** 10.1007/s13132-021-00814-w

**Published:** 2021-07-29

**Authors:** Ewa Cieślik

**Affiliations:** Department of International Economics, Poznan University of Economics and Business, Poznan, Poland

**Keywords:** CEE, Global value chains, Services, ICT services, F14

## Abstract

The article compares changes in tendencies of value-added flows and the places held by the Central and Eastern European economies (11 countries: Visegrad countries, Baltic States, Slovenia, Bulgaria, Croatia, and Romania; CEE) in global value chains (GVCs) in manufacturing, services, and their subgroup—information and telecommunication (ICT) services, by relying on the trade in value-added data retrieved from the OECD’s Inter-Country Input–Output Database, available over the period 2005–2015. The objective of this study is to understand the role of these economies in international production linkages applying a value-added methodology. Therefore, the study discusses the role of CEE economies in global economy in terms of forms of participation of services, especially ICT services, in GVCs, including the process of “servicification” of manufacturing. This study led to the verification of two hypotheses: (1) the position of manufacturing in GVCs has been steadily weakening and (2) services, especially ICT services, can have a positive effect on participation of CEE economies in GVCs. The first hypothesis was confirmed by most of analysed countries. The other research question turned out not to be true for all CEE countries, but most of them proved this hypothesis.

## Introduction

The growing role of services in the world economy is undeniable. Services have long been perceived as non-tradable goods, but technological progress, especially in information and communication technologies (ICT), along with the process of offshoring and development of global value chains (GVCs) has made many types of services increasingly tradeable. This phenomenon is confirmed by the latest World Trade Organization’s data, which highlights that services sector has become the most dynamic component of international trade and that its role has grown in importance. The service sector accounts for about 50% of GDP worldwide, on average. For developed economies, these numbers are much higher—around three-fourths of their GDP is created by services. The services share in GDP proportion is also rapidly growing in developing economies. Moreover, trade in services has been increasing on average 5.4% per year since 2005. In contrast, trade in merchandise has been increasing at a 4.6% pace since 2005 annually. Trade in ICT and R&D services has recorded the most rapid annual growth over the past decade. The organisation predicts that by 2040, the share of trade in services in total trade could increase by 50%. This phenomenon is the result of decreasing trade costs, digitalization process, and lifting restrictions (WTO, 2019). Due to the notable digital and technological developments, the service sector has significantly contributed to productivity growth. Progress, digitalization, and significant reductions in transport costs have made a number of services more tradable. Moreover, automation has changed the labour-intensive manufacturing sectors, and finally, services account for a much higher share of value added than manufacturing; in 2015, services (including construction) accounted for almost 71% of global value added (OECD, 2020).

The perception of services has dramatically changed over time. Instead of being the corollary of manufacturing, services are nowadays among the most dynamic industries in many economies. In the literature, there are two views of the role of services—one view perceives services as a quite separate and independent activity that should be studied apart from manufacturing; the second view connects services to manufacturing, a phenomenon called “servicification” of manufacturing.^1^ Unfortunately, in practice, it is difficult to distinguish between services sold separately and services embodied in other products, e.g., manufacturing goods. This problem is particularly vital in terms of ICT services. Therefore, the article presents the role of services in general (including “servicification” of manufacturing) and services within manufacturing goods.

In connection with the above observations, this paper makes several important contributions to the literature. Firstly, it focuses on flows of value added as the more appropriate method of measuring the role of services in global production based on fragmentation than the standard merchandise flows. Secondly, the importance of cross-sectoral linkages has been demonstrated in many empirical studies at country level (some of them are mentioned in the next section), but there are limited studies on international dimension of cross-sectoral value-added flows (OECD, 2013). Thirdly, there is a gap in the literature regarding research on ICT services. The role of services in trade is widely discussed, but ICT as a subgroup of services have been very rarely tested themselves. Fourthly, Central and Eastern Europe (CEE) as participants of service GVCs are fairly overlooked in research. Analyzing service sector in these economies derives from the fact that the role of this activity in their GDP is increasing as these countries have moved towards more advanced economies. In the flagship industries (automotive and electronics), services have become an integrated part of manufacturing, and what is more, the turnover of services (in particular ICT services) as an individual activity is also vital.

This article compares flows and places held by the CEE economies (11 countries: Visegrad countries—V4, Baltic States, Slovenia, Bulgaria, Croatia, and Romania; EU-11) in GVCs in manufacturing, services, and their subgroup—ICT services, by relying on the trade in value-added data retrieved from the OECD’s Inter-Country Input–Output Database, available over the period 2005–2015 (sometimes till 2016). This database was similar to the World Input–Output Database, but provides the most up-to-date detailed statistics on sectoral input–output flows for the largest number of countries (in comparison to other available databases).^2^ The author assessed the role of these economies in international production linkages using value-added methodology. This study led to the verification of the hypotheses: (1) the position of manufacturing in GVCs has been steadily weakening and (2) services, especially ICT services, can have a positive effect on participation of CEE economies in GVCs.

The study consists of four sections, “Introduction” and “Conclusions.” Firstly, it discusses the role of ICT services and CEE economies in global production linkages in services in the light of the literature, followed by a brief description of the methodology applied in this study. “Method” and “Results” analyse the EU-11 in terms of paths of participation in GVCs. The last section consists of conclusions.

## Literature Review

In this section, a systematic literature review is presented in two parts: in the first part, there is some focus on the general role of ICT services in GVCs, and the second part refers to the CEE economies and their role in GVCs, in particular in terms of the service sector.

### Positioning of the ICT Sector in GVCs

One perceives ICT services’ role in GVCs in three dimensions: as a link that supported GVCs’ development, as an integral part of manufacturing and other services, and as the outsourced content of GVCs. These dimensions interpenetrate one another.

ICT services facilitate the emergence of GVCs in a way that goods do not; they enable (with other services) GVCs to emerge. Production fragmentation was partly driven by services such as ICT, which diminished costs and made it possible to coordinate production in separate stages (Heuser & Mattoo, 2017). Without service links (including ICT services), there would be no GVCs (Jones & Kierzkowski, 1990). As the globalization of production develops, many companies, predominantly from more advanced economies, try to improve their gains and cost efficiency by delegating some part of their activities offshore, either to a captive entity or to a third-party service provider (Gereffi & Fernandez-Stark, 2011). Technological innovations combined with new business models have profoundly altered the nature of services provision and structure for certain categories of services. ICT services have recorded high growth rates of labour productivity over the last decades. Moreover, deregulation and liberalization of service trade have allowed ICT service companies to enter new markets abroad; ICT services are increasingly offshored (Massini & Miozzo, 2012). This process has created opportunities for developing countries to participate in GVCs without having to develop the full range of capabilities of the whole production network. Some studies implied that countries and firms specialized in ICT services segment in GVCs (Cohen et al., 2009 or Contractor et al., 2010). Furthermore, there is greater demand for ICT services to perform skill-intensive tasks, including software development or machine maintenance. Adopting the lean manufacturing paradigm requires firms to spend their resources in the development of ICT services. The rapid growth of e-commerce increases demand for ICT services (Anukoonwattaka et al., 2017).

ICT services are also important inputs of manufacturing and other services. In the concept of “servicification,” services (especially ICT services) allow for the differentiation of manufacturing goods and increase their value. According to empirical research, many economies are more and more often using services in manufacturing as well as selling services directly to the consumers. For example, Lodefalk (2013) argued that manufacturing in industrialised countries is dependent on services more intensively than before. ICT services such as telecommunications and computer services are important inputs to other sectors and are crucial for productivity.

Results from the OECD/WTO partner questionnaire confirm that ICT services are a greater priority for developing countries than ICT manufacturing; e.g., more than half of official development assistance recipients have included ICT services in their development strategies (OECD & WTO, 2013). Miroudot and Cadestin (2017) estimated that the share of total services value added in manufacturing firms across OECD economies was nearly 40%. Neely et al. (2011) proved that by 2011, around 46% companies in Malaysia, 25% in Thailand, and 22% in Indonesia had undergone the “servicification” process. Kox (2004), Pilat and Wölfl (2005), Breinlich and Criscuolo (2011), and Foster et al. (2012) argued that services (including ICT) played an important role in the national innovation system, are responsible for growth of revenues, and create spillovers to other sectors. The growing role of services in manufacturing was raised by Nordås (2010) and Rueda-Cantuche et al. (2012). Šidlauskaitė and Miškinis (2013), Kowalski et al. (2015), and Beverelli et al. (2016) also showed strong linkages (usually forward ones) between services and manufacturing that were reflected in a higher level of GVCs’ integration. Díaz-Mora et al. (2018), Liu et al. (2018), or Blázquez et al. (2020) also found positive impact of services (including ICT) on manufacturing. Francois and Wörz (2008) and Wolfmayr (2008) studied the significance of services on manufacturing competitiveness in OECD countries. Landesmann and Leitner (2015) focused on the EU economies in terms of services’ influence on competitiveness. Gereffi and Fernandez‐Stark (2011) perceived services, especially ICT services, transportation, and logistics as a “glue” in GVCs. In international terms, “servicification” improves the performance of manufacturing firms with more diversified exporting varieties (Kelle et al., 2013) and better access to foreign market (Lodefalk, 2017).

### Positioning CEE Countries in GVCs

Many studies have been conducted with the aim to analyse the CEE economies’ GVCs linkages in the context of an international economy. Suppliers from the CEE region became increasingly integrated into the EU GVCs, as proven by Domanski and Gwóźdź (2009) or Jürgens and Krzywdzinski (2009). There were some signs of upgrading the CEE’s role in GVCs before the crisis (Sass & Szalavetz, 2013; Vlčková et al., 2015; Nunn, 2007; Sankot & Hnát, 2015; Ma et al., 2010 or Cieślik et al., 2016). Nevertheless, this trend did not continue (Cieślik et al., 2019). Many studies examining the case of CEE states have emerged, but the literature suggests that the transportation industry played the predominant role (Sturgeon et al., 2008; Pavlínek & Ženka, 2011; Pavlínek, 2018, 2019). Other sectors have been examined more rarely and selectively, e.g., the apparel industry in Slovakia by Smith et al. (2014) or Plank and Staritz (2013) and electronics in Hungary by Sass and Szalavets (2014).

Smith and Swain (2010) argued that CEE state integration with the EU was driven mainly by the export-led development and GVCs, which was an important implication for the dissemination of the crisis. Kandilov and Grennes (2010) and Marin (2010) examined the changes in employment and labour structure in CEE economies as a result of joining GVCs. Hillberry (2011) proved that improvement in transport connectivity had been significant for production fragmentation intensifications in the CEE countries. Behar and Freund (2011) applied international trade statistics on intermediate products to examine fragmentation in Europe. They discussed how the process of EU integration could facilitate the intra-EU trade in intermediate goods. In turn, Elekdag and Muir (2013) examined the relations between Germany and CEE. They stated that these countries underwent deep economic integration, which had led to the development of GVCs within the EU. They suggested that final demand in Germany was not necessarily the main determinant of the CEE economies’ exports to Germany. Moreover, the study proved that the side effect of the growth in the openness of the analysed countries was a greater exposure of the CEE states to global shocks.

There were also some pieces of literature proving that Germany, in particular, gradually weakened its trade ties with Southern Europe in favour of CEE states (Simonazzi et al., 2013). Similar conclusions were drawn by Coricelli and Wörgötter (2012), who argued that for Germany, the New Members of the EU were “an opportunity to outsource low-skilled processes abroad, import the necessary inputs from Central European cost-efficient economies and keep the mid-skilled processes on domestic soil.” There were some signs of upgrading the CEE’s role in GVCs before the crisis (Sass & Szalavetz, 2013), however.

According to some studies (Fortwengel, 2011; Cieślik et al., 2019; Jacoby, 2010; Dobrinsky, 1995 or Vrh, 2015), the CEE economies are mainly found in downstream markets in GVCs. The CEE states’ exports, expansion, and competitiveness have been supported by acquired technology, capital, and know-how. Although CEE countries became an important part of GVCs before the crisis, now they are losing their position even in their flagship industries.

There are very few studies focusing on the role of services in GVCs of CEE economy. The EU economy is most often portrayed in such studies, but CEE countries are depicted occasionally. Partly the role of services in CEE’s connection to the world production chain was presented in studies by Pellenyi (2020) and Cieślik (2019). Moreover, this research was conducted at least a decade ago. Usually, this participation was analysed with regards to manufacturing or as a background to manufacturing, especially in automotive or electronics industries. Many authors did not treat services as an important part of GVCs in the CEE states before the crisis. Nowadays, this perception has changed, while the CEE economies are losing their position in manufacturing GVCs, in particular in their flagship industries. Therefore, this article is a contribution to the literature on the subject. An evaluation of the CEE economies’ participation and relative positions in GVCs, as well as the foreign value added embodied in their gross exports and domestic value added embodied in their trade partners’ gross trade, has never been conducted, to the best of the author’s knowledge.

## Method

To analyse the participation and position of the CEE economy in GVCs, the methodology of value-added flow was applied. A multi-regional input–output model which also included value added in industries/sectors was developed. This approach was a combination of methods adopted by several authors: Hummels et al. (2001), Johnson and Noguera (2012), Koopman et al. (2014), and Timmer et al. (2013). The above-mentioned authors applied models for calculating the added value in relation to the entire economy, and not to individual sectors. As part of the approach used in the monograph, the above-mentioned models were extended to include sector/industry analyses, to the point statistical data allowed.

Using this method, it is possible to answer the question of whether a country being located in an upstream segment in the production value chain (first stage of production) influences the likeliness of it having a high value of forward links relative to backward ones. It means that the country relies more on its own production. If a country specializes in the final stages of production (downstream segment), it is likely that it imports a lot of intermediate goods from abroad, and therefore, it has high backward participation.

**Fig. 1:**
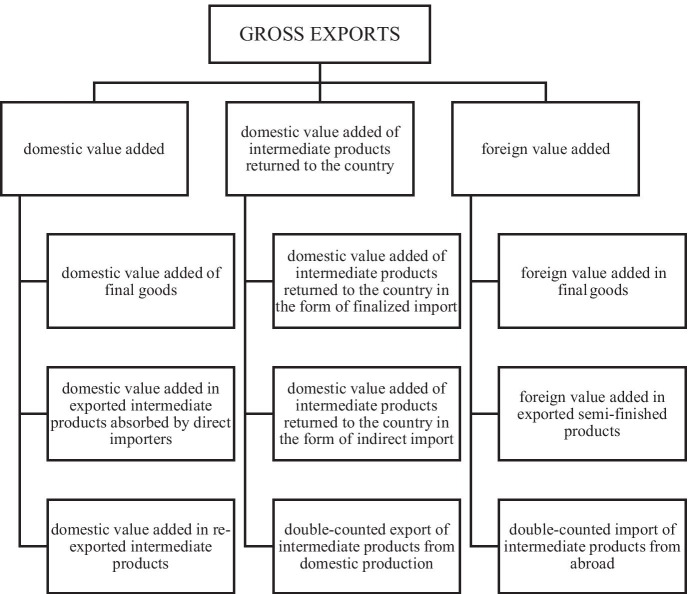
Diagram of gross export decomposition. Source: Own elaboration based on Koopman et al., 2014

The key to calculating the foreign value added embodied in gross exports (FVA) and domestic value added embodied in gross exports (IVA) is to define what they are and what distinguishes them from standard calculations—subject to imperfection related to the so-called “double counting” in foreign trade. In order to remove this effect from the *aggregated statistics* on *trade*, gross export was decomposed and three categories were distinguished therein: domestic added value, domestic added value in indirect exports which returns to the home country, and foreign added value. Details of this division are presented in Fig. 1.

The approach assumes that in each economy, there are S sectors/industries in the number of N economies. Each sector/industry is responsible for producing one differentiated good. Production (x) is used both to meet the final demand (y) and to produce intermediate goods (semi-finished products) marked with the symbol z, which will then be used in the further process of manufacturing products both domestically and abroad.

X_i_ (s) = Σ_j_y_ij_ (s) + Σ_j_Σtz_ij_ (s, t)

There can be defined A matrix with dimensions SN × SN, with elements.

a_ij_ (s, t) = z_ij_ (s,t)/x_j_ (t)

Thus, the basic condition was formulated.

x = (I − A)^−1^y

This can be represented in the form of the matrix below:

$$\left[\begin{array}{c}{X}_{1}\\ \vdots \\ {X}_{N}\end{array}\right]={\left[\begin{array}{ccc}I-{A}_{11}& \cdots & -{A}_{1N}\\ \vdots & \ddots & \vdots \\ -{A}_{N1}& \cdots & I-{A}_{NN}\end{array}\right]}^{-1}\left[\begin{array}{c}\sum_{i}^{N}{Y}_{1j}\\ \vdots \\ \sum_{i}^{N}{Y}_{Nj}\end{array}\right]={\left[\begin{array}{ccc}{Z}_{11}& \cdots & {Z}_{1N}\\ \vdots & \ddots & \vdots \\ {Z}_{N1}& \cdots & {Z}_{NN}\end{array}\right]}^{-1}\left[\begin{array}{c}{Y}_{1}\\ \vdots \\ {Y}_{N}\end{array}\right]$$

The matrix can be transformed into the following input–output model:

$$\left[\begin{array}{ccc}{X}_{11}& \cdots & {X}_{1N}\\ \vdots & \ddots & \vdots \\ {X}_{N1}& \cdots & {X}_{NN}\end{array}\right]=\left[\begin{array}{ccc}{Z}_{11}& \cdots & {Z}_{1N}\\ \vdots & \ddots & \vdots \\ {Z}_{N1}& \cdots & {Z}_{NN}\end{array}\right]\left[\begin{array}{ccc}{Y}_{11}& \cdots & {Y}_{1N}\\ \vdots & \ddots & \vdots \\ {Y}_{N1}& \cdots & {Y}_{NN}\end{array}\right]$$

where:

N—number of countries, S-number of sectors, Z—matrix with SNxSN dimension, X and Y—matrices;

Z—gross production in i economy needed to meet the final demand in j economy;

X—output produced in i country and absorbed by j country’s market;

Y—output produced in the i country and absorbed by j country;

Then the production matrix with added value $\widehat{V}ZY$ was formulated

$$\begin{bmatrix}{\widetilde V}_1&\cdots&0\\\vdots&\ddots&\vdots\\0&\cdots&{\widetilde V}_N\end{bmatrix}\begin{bmatrix}X_{11}&\cdots&X_{1N}\\\vdots&\ddots&\vdots\\X_{N1}&\cdots&X_{NN}\end{bmatrix}=\begin{bmatrix}{{\widehat V}_1{\textstyle\sum_J^N}Z}_{1j}Y_{j1}&\cdots&{{\widehat V}_1{\textstyle\sum_J^N}Z}_{1j}Y_{jN}\\\vdots&\ddots&\vdots\\{{\widehat V}_N{\textstyle\sum_J^N}Z}_{Nj}Y_{j1}&\cdots&{{\widehat V}_N{\textstyle\sum_J^N}Z}_{Nj}Y_{jN}\end{bmatrix}$$

The diagonal matrix represents the value-added of production that has been absorbed by the country, while the elements outside the diagonal matrix represent the value-added absorbed abroad (value added embodied in the trade partner’s gross exports).

$$\begin{aligned}VAE_i& ={\textstyle\sum_{j\neq i}^N}VX_{ij}=V_i{\textstyle\sum_{j\neq i}^N}{\textstyle\sum_{n=1}^N}Z_{in}Y_{nj} =Vi{\textstyle\sum_{j\neq i}^N}Z_{ii}Y_{ij}\\ &+V_i{\textstyle\sum_{j\neq i}^N}Z_{ij}Y_{jj}+V_i{\textstyle\sum_{j\neq i}^N}{\textstyle\sum_{t\neq i}^N}Z_{ij}Y_{jt}\end{aligned}$$  

Where:

${\mathrm{V}}_{\mathrm{i}}{\sum }_{j\ne i}^{N}{Z}_{ii}{Y}_{ij}$—value added embodied (included) in gross exports of final goods;

${V}_{i}\sum_{j\ne 1}^{N}{Z}_{ij}{Y}_{jj}$—value added embodied (included) in gross exports of intermediate goods (semi-finished goods);

${V}_{i}\sum_{j\ne i}^{N}\sum_{t\ne ij}^{N}{Z}_{ij}{Y}_{jt}$—indirect value added embodied (included) in gross exports.

In order to formulate the share of foreign value added in the gross export of the analysed country, the following notation must be used:

$$\begin{aligned}VS &={\textstyle\sum_{j\neq i}^N}V_jZ_{ji}E_{i\ast}={\textstyle\sum_{t\neq i}^N}{\textstyle\sum_{j\neq i}^N}V_tZ_{ti}Y_{ij} \\ &+{\textstyle\sum_{t\neq i}^N}{\textstyle\sum_{j\neq i}^N}V_tZ_{ti}A_{ij}{(I-A_{jj})}^{-1}Y_{ij} \\ &+{\textstyle\sum_{j\neq i}^N}V_tZ_{ti}{(I-A_{jj})}^{-1}E_{j\ast}\end{aligned}$$  

Where:

$\sum_{t\ne i}^{N}\sum_{j\ne i}^{N}{{V}_{t}Z}_{ti}{Y}_{ij}$—foreign value added included in gross exports of final goods;

${\sum }_{t\ne i}^{N}{\sum }_{j\ne i}^{N}{V}_{t}{Z}_{ti}{A}_{ij}{\left(I-{A}_{jj}\right)}^{-1}{Y}_{jj}$—foreign value added in gross exports of intermediate goods;

$\sum_{j\ne i}^{N}{V}_{t}{Z}_{ti}{\left(I-{A}_{jj}\right)}^{-1}{E}_{j*}$—double value added of intermediate goods produced abroad.

Finally, the export of intermediate goods, which is used as an input to the production of goods abroad, can be presented as follows:

$$\begin{aligned}VSI & =V_i{\textstyle\sum_{j\neq i}^N}Z_{ji}E_{i\ast}=V_i{\textstyle\sum_{t\neq ij}^N}{\textstyle\sum_{j\neq i}^N}Z_{ij}Y_{jt}\\ &+V_i{\textstyle\sum_{t\neq ii}^N}{\textstyle\sum_{j\neq i}^N}Z_{ij}A_{ij}A_{jt}X_t \\ &+V_i{\textstyle\sum_{t\neq ij}^N}{\textstyle\sum_{j\neq i}^N}Z_{ij}Y_{ji}\\ &+V_i{\textstyle\sum_{j\neq ij}^N}Z_{ij}A_{ji}X_i\end{aligned}$$  

Where:

${V}_{i}\sum_{t\ne ij}^{N}\sum_{j\ne i}^{N}{Z}_{ij}{Y}_{jt}$—indirect value added embodied in gross exports;

${V}_{i}\sum_{t\ne ij}^{N}\sum_{j\ne i}^{N}{Z}_{ij}{A}_{ij}{A}_{jt}{X}_{t}$—exports of intermediate goods that will be used abroad to produce gross exports of intermediate goods;

${V}_{i}\sum_{t\ne ij}^{N}\sum_{j\ne i}^{N}{Z}_{ij}{Y}_{ji}$—domestic value added that returns to the country in the form of final products;

${V}_{i}\sum_{j\ne i}^{N}{Z}_{ij}{A}_{ji}{X}_{i}$—domestic value added that returns through import of intermediate goods.

Finally, a decomposition of gross exports can be formulated in the form of:

$$\begin{aligned}DCP& =\lbrack V_i\ {\textstyle\sum_{j\neq i}^N}Z_{ij}Y_{ij}+V_i\ {\textstyle\sum_{j\neq1}^N}Z_{ij}Y_{jj}+V_i\ {\textstyle\sum_{j\neq i}^N}{\textstyle\sum_{t\neq ij}^N}Z_{ij}Y_{jt}\rbrack \\ &+[{\textstyle\sum_{t\neq i}^N}{\textstyle\sum_{j\neq i}^N}V_tZ_{ti}Y_{ij}+{\textstyle\sum_{t\neq i}^N}{\textstyle\sum_{j\neq i}^N}V_tZ_{ti}A_{ij}{(I-A_{jj})}^{-1}Y_{jj}\\ &+{\textstyle\sum_{j\neq i}^N}V_tZ_{ti}{(I-A_{jj})}^{-1}E_{j\ast}]+ \lbrack V_i{\textstyle\sum_{t\neq ij}^N}{\textstyle\sum_{j\neq i}^N}Z_{ij}Y_{ji}\\ &+V_i{\textstyle\sum_{t\neq ij}^N}{\textstyle\sum_{j\neq i}^N}Z_{ij}A_{jt}{(I-A_{ii})}^{-1}Y_{ii}+V_i{\textstyle\sum_{j\neq i}^N}Z_{ij}A_{jt}{(I-A_{ii})}^{-1}E_{i\ast}\end{aligned}$$  

The above formulas can be explained as follows:

${\mathrm{V}}_{i}\sum_{j\ne i}^{N}{Z}_{ii}{Y}_{ij}+{V}_{i}\sum_{j\ne 1}^{N}{Z}_{ij}{Y}_{jj}+{V}_{i}\sum_{j\ne i}^{N}\sum_{t\ne ij}^{N}{Z}_{ij}{Y}_{jt}$ and ${\mathrm{V}}_{\mathrm{i}}\sum_{t\ne ij}^{N}\sum_{j\ne 1}^{N}{Z}_{ij}{Y}_{jj}+{V}_{i}\sum_{t\ne ij}^{N}\sum_{j\ne i}^{N}{Z}_{ij}{A}_{ji}{\left(I-{A}_{ii}\right)}^{-1}{Y}_{ii}+{V}_{i}\sum_{j\ne i}^{N}{Z}_{ij}{A}_{jt}{\left(I-{A}_{ii}\right)}^{-1}{E}_{1*}$—domestic value added embodied (included) in the trade partner's gross exports (IVA).

$\sum_{t\ne i}^{N}\sum_{j\ne 1}^{N}{V}_{t}{Z}_{ti}{Y}_{ij}+\sum_{t\ne i}^{N}\sum_{j\ne i}^{N}{V}_{t}{Z}_{ti}{A}_{ij}{\left(I-{A}_{jj}\right)}^{-1}{Y}_{jj}+\sum_{j\ne i}^{N}{V}_{t}{Z}_{ti}{\left(I-{A}_{jj}\right)}^{-1}{E}_{1*}$—foreign value added embodied (included) in the country's gross exports (FVA).

The above-presented formulas for calculating the share of domestic value added embodied (included) in gross exports of partner-countries (IVA) and foreign value added embodied (included) in gross exports of the country (FVA) also allow determining the country's relations with the foreign countries. Therefore, IVA means the so-called forward linkages in GVCs, while FVA can be equated with the so-called backward linkages.

Using the method described above, it is possible to construct an index that allows indicating the position of a country in GVCs in relation to other countries and assessing whether the country is in the *upstream/downstream market / segment*. A country that is in the upstream market produces goods used by other countries in their gross exports (economy exports domestic products and intermediate goods to foreign partners responsible for the next stages of production—*forward linkages*). A country that is in the downstream market uses intermediate goods manufactured in other economies to produce final goods, which are then exported to that country’s trading partners (the economy participates in the GVCs by importing intermediate goods from abroad, which it then uses to produce exported goods and services—*backward linkages*).

The following index, which measures the relative position of a country or sector, may be obtained by taking into account the relative importance of obtaining production resources and processing production.

GVC_POSITION = Ln (1 + IVA) − Ln (1 + FVA),

The relatively higher value of the FVA places the country in the *downstream* market, and therefore, the values of the calculated index are negative. On the contrary, when a relatively higher IVA value is observed, the country is in the *upstream* market, and thus, the index values are positive. A similar analysis can be performed for a specific sector/industry.

## Results

Because of the transformation, as well as the growth of integration between the CEE economy and the EU countries, the CEE states entered not only merchandise GVCs, but also service linkages recently. The evolution of CEE and EU plans to invite these countries encouraged EU-based companies to invest in reducing production costs, particularly labour costs, and to exploit the comparative advantages (so-called efficiency-seeking investments). Certainly, lower labour costs compensated the considerably lower productivity of employees in the CEE economy compared with the EU-15. For many investors, these markets become the extension of the domestic market and their production linkages. Furthermore, these countries themselves were perceived as sufficient and very attractive locations for the EU’s FDI.

Unfortunately, the streak was short-lived and the EU-11 started to deteriorate their relative position in manufacturing GVCs. Moreover, these economies have faced strong competition from Asian developing countries, in particular China that are determined to climb the manufacturing-product linkages and are developing capabilities in knowledge-intensive manufacturing. Therefore, the CEE countries need a new activity that will be competitive and raise them up into the upstream market in GVCs. This activity could be a service sector, especially more advanced services. According to the OECD’s latest available data, Latvia and Croatia have been characterized by the largest share of the service sector in GDP (above 70%). In Poland, services created only 57% of GDP, which was the lowest result among the analysed states. Nevertheless, all these countries’ national production relied mostly on the service sector (OECD, 2020).

The EU-11 was mostly characterized by a significantly high share of FVA in the examined years. After the global crisis in 2008, FVA in total gross exports fluctuated significantly. In all analysed countries, except for Croatia, it peaked in 2014 and then fluctuated or sometimes showed a downward tendency. In 2016, Croatia was characterized by the lowest FVA (19.17% of gross exports) and Slovakia relied the most on FVA (44.51%). The statistics show that the FVA surged in Estonia (4.06% growth between 2005 and 2015) significantly and fell dramatically in Romania (5.93% decrease). It is worth noting that the vast majority of countries experienced an increase in FVA, which is not an optimistic picture (Table 1).

**Table 1:** CEEs in GVCs in selected years

		FVA (% of gross exports)					
		2005	2009	2011	2014	2015	**2016**
Czechia	Total	34.43	39.39	45.09	46.61	39.28	37.67
	Manufacturing	40.60	40.05	45.26	45.59	46.03	44.05
	Services	17.70	17.19	20.44	22.04	19.76	19.48
	Services to total	0.51	0.44	0.45	0.47	0.50	0.52
Hungary	Total	44.01	39.91	48.48	47.31	43.10	44.14
	Manufacturing	52.32	53.98	58.12	56.03	51.29	53.99
	Services	16.78	19.23	21.11	21.87	23.41	21.37
	Services to total	0.38	0.48	0.44	0.46	0.54	0.48
Poland	Total	24.68	27.89	32.29	32.98	26.64	26.90
	Manufacturing	32.01	31.71	36.55	35.57	34.29	34.78
	Services	13.15	12.49	14.38	13.74	14.00	14.47
	Services to total	0.53	0.45	0.45	0.42	0.53	0.54
Slovakia	Total	42.99	44.35	46.73	48.19	44.78	44.51
	Manufacturing	51.67	51.14	54.80	53.81	52.36	52.60
	Services	20.45	15.75	18.88	19.50	20.27	20.74
	Services to total	0.48	0.36	0.40	0.40	0.45	0.47
Estonia	Total	30.41	27.82	38.41	36.94	34.81	34.47
	Manufacturing	39.06	37.82	49.64	48.17	45.57	46.40
	Services	22.77	18.56	23.33	23.49	22.55	21.36
	Servicesto total	0.75	0.67	0.61	0.64	0.65	0.62
Latvia	Total	21.37	18.75	23.24	22.20	22.36	20.74
	Manufacturing	28.35	24.82	31.01	29.58	29.67	28.61
	Services	15.69	15.09	16.59	16.01	16.70	14.97
	Services to total	0.73	0.80	0.71	0.72	0.75	0.72
Lithuania	Total	29.49	26.81	35.46	32.58	31.60	29.43
	Manufacturing	40.87	39.85	48.08	43.48	42.89	40.71
	Services	13.83	11.83	15.71	18.17	17.28	16.61
	Services to total	0.47	0.44	0.44	0.56	0.55	0.56
Bulgaria	Total	32.40	31.72	37.16	37.27	36.24	32.19
	Manufacturing	45.51	43.27	48.56	49.55	48.36	43.88
	Services	23.32	19.32	18.56	21.33	20.90	19.09
	Services to total	0.72	0.61	0.50	0.57	0.58	0.59
Croatia	Total	22.31	19.01	19.44	19.02	20.04	19.17
	Manufacturing	31.59	27.73	28.50	27.80	29.37	28.44
	Services	15.80	13.18	12.80	13.06	13.63	13.26
	Services to total	0.71	0.69	0.66	0.69	0.68	0.69
Romania	Total	27.56	19.77	23.67	23.59	22.92	21.63
	Manufacturing	33.71	25.00	25.73	28.82	29.85	27.43
	Services	13.37	12.76	21.48	17.51	14.69	15.41
	Services to total	0.49	0.65	0.91	0.74	0.64	0.71
Slovenia	Total	33.28	37.52	34.40	36.11	32.46	31.55
	Manufacturing	40.64	37.82	43.04	41.41	39.32	39.36
	Services	16.85	16.91	19.51	19.47	19.49	18.29
	Services to total	0.51	0.45	0.57	0.54	0.60	0.58

		IVA (% of gross exports)					
		2005	2009	2011	2014	2015	2016
Czechia	Total	25.52	23.00	23.35	21.30	22.20	21.96
	Manufacturing	25.41	25.13	22.71	21.25	21.79	na
	Services	25.59	28.07	26.50	23.84	23.34	na
	Services to total	1.00	1.22	1.13	1.12	1.05	na
Hungary	Total	19.91	18.70	19.07	18.38	14.72	14.74
	Manufacturing	18.29	17.31	14.68	14.50	13.88	na
	Services	25.41	22.34	19.39	17.60	16.85	na
	Services to total	1.28	1.19	1.02	0.96	1.14	na
Poland	Total	31.45	20.50	29.65	28.57	28.45	28.01
	Manufacturing	34.93	32.96	32.99	32.08	30.66	na
	Services	26.07	25.97	23.86	23.56	24.35	na
	Services to total	0.83	1.27	0.80	0.82	0.86	na
Slovakia	Total	17.90	17.90	21.49	20.14	19.54	18.79
	Manufacturing	17.17	21.00	18.43	19.10	19.09	na
	Services	19.60	20.15	21.07	19.65	21.29	na
	Services to total	1.09	1.13	0.98	0.98	1.09	na
Estonia	Total	23.54	24.22	20.98	22.11	21.70	21.29
	Manufacturing	25.94	26.20	21.76	23.20	22.24	na
	Services	21.37	22.25	19.55	20.73	21.29	na
	Services to total	0.91	0.92	0.93	0.94	0.98	na
Latvia	Total	26.17	28.25	26.51	27.94	28.43	27.44
	Manufacturing	33.46	36.92	31.33	31.98	32.18	na
	Services	20.19	23.38	22.54	24.46	25.55	na
	Services to total	0.77	0.83	0.85	0.88	0.90	na
Lithuania	Total	18.45	22.78	17.32	16.85	17.71	16.04
	Manufacturing	20.32	23.75	17.42	19.08	18.66	na
	Services	15.33	20.31	15.95	12.39	15.90	na
	Services to total	0.83	0.89	0.92	0.74	0.90	na
Bulgaria	Total	24.47	25.80	25.90	23.42	23.44	24.84
	Manufacturing	25.44	27.48	25.75	23.29	23.21	na
	Services	24.82	24.28	27.36	24.95	24.92	na
	Services to total	1.01	0.94	1.06	1.07	1.06	na
Croatia	Total	23.73	25.03	25.86	25.13	24.51	24.50
	Manufacturing	27.81	30.34	31.45	30.13	28.39	na
	Services	21.17	21.79	21.93	21.88	22.00	na
	Services to total	0.89	0.87	0.85	0.87	0.90	na
Romania	Total	23.94	25.82	28.95	32.35	27.48	28.34
	Manufacturing	25.19	26.87	26.10	32.41	26.60	na
	Services	21.31	24.33	36.22	33.16	28.67	na
	Services to total	0.89	0.94	1.25	1.03	1.04	na
Slovenia	Total	21.48	18.20	24.62	23.91	21.06	20.35
	Manufacturing	21.28	23.49	21.19	20.50	20.09	na
	Services	22.02	22.15	21.73	21.06	22.84	na
	Services to total	1.03	1.22	0.88	0.88	1.08	na

		Relative position in GVCs					
		2005	2009	2011	2014	2015	2016
Czechia	Total	−0.07	−0.13	−0.16	−0.19	−0.13	−0.12
	Manufacturing	−0.11	−0.11	−0.17	−0.18	−0.18	na
	Services	**0.06**	**0.09**	**0.05**	**0.01**	**0.03**	na
	Services to total						
Hungary	Total	−0.18	−0.16	−0.22	−0.22	−0.22	−0.23
	Manufacturing	−0.25	−0.27	−0.32	−0.31	−0.28	na
	Services	**0.07**	**0.03**	−0.01	−0.04	−0.05	na
	Services to total						
Poland	Total	**0.05**	−0.06	−0.02	−0.03	**0.01**	**0.01**
	Manufacturing	**0.02**	**0.01**	−0.03	−0.03	−0.03	na
	Services	**0.11**	**0.11**	**0.08**	**0.08**	**0.09**	na
	Services to total						
Slovakia	Total	−0.19	−0.20	−0.19	−0.21	−0.19	−0.20
	Manufacturing	−0.26	−0.22	−0.27	−0.26	−0.25	na
	Services	−0.01	**0.04**	**0.02**	**0.00**	**0.01**	na
	Services to total						
Estonia	Total	−0.05	−0.03	−0.13	−0.11	−0.10	−0.10
	Manufacturing	−0.10	−0.09	−0.21	−0.18	−0.17	na
	Services	−0.01	0.03	−0.03	−0.02	−0.01	na
	Services to total						
Latvia	Total	**0.04**	**0.08**	**0.03**	**0.05**	**0.05**	**0.05**
	Manufacturing	**0.04**	**0.09**	**0.00**	**0.02**	**0.02**	na
	Services	**0.04**	**0.07**	**0.05**	**0.07**	**0.07**	na
	Services to total						
Lithuania	Total	−0.09	−0.03	−0.14	−0.13	−0.11	−0.11
	Manufacturing	−0.16	−0.12	−0.23	−0.19	−0.19	na
	Services	**0.01**	**0.07**	0.00	−0.05	−0.01	na
	Services to total						
Bulgaria	Total	−0.06	−0.05	−0.09	−0.11	−0.10	−0.06
	Manufacturing	−0.15	−0.12	−0.17	−0.19	−0.19	na
	Services	**0.01**	**0.04**	**0.07**	**0.03**	**0.03**	na
	Services to total						
Croatia	Total	**0.01**	**0.05**	**0.05**	**0.05**	**0.04**	**0.04**
	Manufacturing	−0.03	0.02	0.02	0.02	−0.01	na
	Services	**0.05**	**0.07**	**0.08**	**0.08**	**0.07**	na
	Services to total						
Romania	Total	−0.03	**0.05**	**0.04**	**0.07**	**0.04**	**0.05**
	Manufacturing	−0.07	**0.01**	0.00	**0.03**	−0.03	na
	Services	**0.07**	**0.10**	**0.11**	**0.13**	**0.12**	na
	Services to total						
Slovenia	Total	−0.09	−0.15	−0.08	−0.09	−0.09	−0.09
	Manufacturing	−0.15	−0.11	−0.17	−0.16	−0.15	na
	Services	**0.04**	**0.04**	**0.02**	**0.01**	**0.03**	na
	Services to total						

If the relative position in GVCs is higher than 0, it means that the country is located in upstream market. If the relative position in GVCs is lower than 0, it means that the country is located in downstream market. Source: Calculated by the author based on OECD's Inter-Country Input–Output Database, 2020

*na* not available

In the case of total IVA of the EU-11, it is difficult to indicate a uniform trend. Recently, most of the analysed countries have reduced their total IVA or have remained at a similar level. The exceptions were Slovakia, Latvia, Bulgaria, Croatia, and Romania, where IVA grew between 2005 and 2016. The largest reduction of IVA was observed in Hungary (5.17% between 2005 and 2016), which had the lowest IVA share in gross exports in the analysed period (14.74% of gross exports). In Romania, there was the most significant climb in IVA (4.4% between 2005 and 2016). In 2016, the highest IVA was observed in Romania (28.34%) (Table 1).

There is a significant difference between the dependence of services and manufacturing on FVA in CEE. In 2016, the largest gap (difference) between manufacturing and service FVA was noticed in Hungary (32.62%) and Slovakia (31.86%), where manufacturing dominated. The lowest contrast in FVA was in Romania (12.02%). The service sector is not only less dependent on FVA, but also shows no significant increases between 2005 and 2015. The most significant growth in service FVA was observed in Hungary (4.59%), the most noticeable reduction in FVA—in Bulgaria (4.23%). In 2016, a low level of FVA in services persisted in Croatia (13.26%), followed by Poland (14.47%) and Latvia (14.97%). In 2016, Hungary was characterized the largest dependence of FVA in services (21.37%) (Table 1).

The situation becomes more ambiguous when analysing IVA in services. In the vast majority of countries (Romania, Latvia, Croatia, Slovakia, Slovenia, Lithuania and Bulgaria) there was noticed an increase in IVA in services. In terms of manufacturing IVA, there was a similar situation as in total IVA—most of CEE countries reduced their IVA significantly between 2005 and 2015. Only Slovakia, Romania, and Croatia raised their IVA in manufacturing (1.92%, 1.41%, and 0.58%, respectively). Generally, the decline of IVA in UE-11’s manufacturing was much more noticeable than its drop in services (Table 1).

The FVA and IVA values are directly reflected in the relative position in GVCs. In terms of total production, the position of CEE most countries deteriorated, except for Romania, Croatia, and Latvia. Most economies are located in the downstream market. The only exceptions are Poland (minimally in the upstream market), Latvia, Croatia, and Romania. Poland was in the upstream market right after its accession to the EU, but with time it gradually began to lose its importance as a value-added supplier for its trade partners, and it relied more on FVA, mainly from Germany. Latvia and Croatia were in the upstream market throughout the entire period. An interesting case, however, is Romania, which when entering the EU was considered one of the least developed countries (along with Bulgaria). At the time of accession to the EU, the country was located low in the downstream market, but every year it climbed up to be rated next to Latvia (the highest among the CEE countries in relative position in GVCs; 0.05). In 2016, in total production, Hungary placed the lowest. As mentioned earlier, Romania and Latvia positioned the highest. Some of the analysed countries did not change their positions (Slovenia and Bulgaria) (Table 1).

Considering the manufacturing in 2015, almost all CEE countries, except for Latvia, were in the downstream market. The lowest position was held by Hungary and the highest – the aforementioned Latvia. All analysed economies, except for Croatia and Romania, suffered a deterioration in their position. Even Latvia ranked higher in 2005. In terms of services, most of CEE economies were located in the upstream markets in the analysed years (Czechia, Poland, Slovenia, Latvia, Bulgaria, Croatia and Romania). In other states, the situation was changing depending on the year. Hungarian, Estonian, and Lithuanian services were in the least favourable situation and positioned in the downstream market. Comparing the fluctuations of relative positions in GVCs in services to manufacturing, there were lesser standard deviations. Plausible services were responsible for the upward market relative position in total production in Romania, Croatia, or Poland, although manufacturing in these countries ranked in the downstream markets. Unfortunately, there were few cases where the relative positions in the services GVCs of CEE countries increased between 2005 and 2015 (Slovakia, Latvia, Bulgaria, Croatia, and Romania); in other economies, these positions deteriorated in this period. Nevertheless, the service sector seems to be much more promising to strengthen the CEE countries’ total position in global production linkages than manufacturing (Table 1).

Due to the great importance of services in shaping the position of EU-11 in GVCs, it was decided to analyse this sector in greater detail. ICT services have occupied an important position among the business services in the CEE economy, and therefore, they will be discussed thoroughly in this section. This study classified ICT services for OECD’s ISIC Revision 4. They consist of publishing, audio-visual and broadcasting activities, telecommunications and IT, and other information services. The study focused on the main category and its subsectoral level, excluding audio-visual and broadcasting activities (they do not play an important role in the category). Several factors influenced the choice of ICT services for this analysis.

Firstly, between 2005 and 2015, ICT services in all CEE countries were characterized by the highest growth of total gross export, gross exports of final products, value added, and gross export of intermediate products. Secondly, after the 2008 crisis, the average productivity growth rate of ICT services in all countries not only outstripped other types of services in most CEE economies, but also exceeded the average annual productivity growth in manufacturing. Moreover, the gross value added per person employed in ICT services also surpassed other service activities after the financial crisis. Thirdly, all countries have introduced programs promoting the development of industry 4.0, including ICT services and innovative technologies.^3^ Fourthly, in all analysed countries, the market value of ICT services has grown significantly. Fifthly, there is an increasing expenditure on R&D reflected in the increasing export of high-tech goods, revenue from the sale of licenses, and patents abroad, as well as the number of registered trademarks and designs (CEIC, 2020).

In most analysed countries, an upward tendency in FVA in ICT services was clearly visible (in V4, Latvia, Lithuania, and Slovenia). Countries that have reduced their dependence on FVA have also become important outsourcing centres for IT support services in Europe (including Romania, Bulgaria) (Table 2). After accession to the EU, V4 absorbed most of FDI related to outsourcing. Despite that, the CEE countries gained importance after the 2008 crisis, which was clearly visible in higher relative positions in GVCs, whereas such a phenomenon did not occur in ICT services (except for Lithuania, Romania, and Bulgaria which introduced special and efficient policies toward innovation).

**Table 2:** ICT services and their subgroups in GVCs in 2005–2015

	FVA (% of gross exports)										
	2005	2006	2007	2008	2009	2010	2011	2012	2013	2014	2015
Czechia											
ICT services:	17.05	18.47	17.52	16.95	17.25	19.78	18.95	19.8	20.36	20.23	19.15
Telecommunications	13.38	13.85	13.61	13.61	13.92	15.77	16.71	18.26	22.16	23.25	24.01
IT and other information services	16.24	16.94	17.75	16.25	16.43	18.81	17.87	18.17	17.88	17.28	15.19
Estonia											
ICT services:	23.24	20.64	18.54	17.85	16.97	20.57	22.1	21.12	21.51	22.17	21.14
Telecommunications	25.5	23.45	20.9	21.09	20.83	26.17	29.18	29.31	29.69	26.55	26.74
IT and other information services	21.59	17.94	15.93	13.26	11.71	13.64	14.71	14.98	16.11	19.95	18.09
Hungary											
ICT services:	14.54	15.88	16.42	17.48	18.39	17.56	19.01	18.8	18.59	18.67	20.69
Telecommunications	12.15	14.2	14.69	14.68	15.61	15.93	16.56	17.44	19.15	19.67	22.81
IT and other information services	15.57	16.57	17.21	17.52	17.66	16.69	16.4	16.1	15.49	14.93	18.9
Latvia											
ICT services:	10.98	13.58	13.66	11.45	11.77	13.15	13.1	14.31	13.68	11.74	12.68
Telecommunications	10.22	12.16	11.91	11.64	12.66	12.68	13.15	15.57	13.39	14.56	15.85
IT and other information services	10.97	14.63	14.16	9.98	8.97	12.05	11.71	12.22	12.48	8.85	10.25
Lithuania											
ICT services:	9.64	10.11	10.87	12.84	9.76	12.16	12.8	12.92	10.95	11.51	12.97
Telecommunications	9.62	10.23	10.41	9.14	8.11	10.25	11.5	11.36	9	11.12	13.29
IT and other information services	8.75	8.71	10.09	21.11	13.06	12.7	13.66	13.04	10.12	10.52	11.74
Poland											
ICT services:	12.46	13.91	13.98	14.27	13.63	15.23	15.49	14.49	13.99	15	15.22
Telecommunications	12.01	13.8	15.41	15.9	14.73	16.02	15.99	14.48	15.41	15.51	17.16
IT and other information services	12.51	13.69	13.75	13.68	13.18	14.65	15	13.89	12.95	13.79	14.25
Slovakia											
ICT services:	21.55	21.27	19.4	17.08	15.27	14.5	13.45	13.59	17.01	18.76	22.29
Telecommunications	15.82	19.16	20.17	14.86	14.06	10.53	9.47	11.74	14.17	15.71	20.98
IT and other information services	21.17	19.85	17.6	17.34	14.49	12.31	12.28	13.37	17.52	20.06	22.96
Slovenia											
ICT services:	18.92	19.13	19.79	19.06	19.68	20.72	21.05	22.08	22.58	21.88	22.56
Telecommunications	18.9	19.32	19.36	19.52	21.25	22.43	22.8	24.33	25.17	24.11	25.36
IT and other information services	15.75	15.09	15.67	15.04	15.6	16.14	15.44	16.72	16.25	15.13	16.07
Bulgaria											
ICT services:	20.49	19.77	19.37	14.13	15.99	14.14	11.62	14.85	12.54	14.41	17.3
Telecommunications	19.92	19.17	18	9.35	13.78	12.84	11.07	16.27	12.76	16.42	18.29
IT and other information services	22.36	19.63	17.72	16.39	17.27	11	9.62	12.25	10.42	13.07	16.8
Croatia											
ICT services:	16.35	15.33	15.55	14.53	13.35	12.11	13.04	13.02	13.55	13.55	13.7
Telecommunications	12.94	13.39	13.97	11.71	10.46	9.43	11.78	12.18	12.97	12.96	13.71
IT and other information services	15.66	15.48	13.72	13.54	12.85	9.9	11.06	11.4	11.98	12.41	11.92
Romania											
ICT services:	13.92	13.78	11.98	10.97	11.39	16.07	16.69	14.58	13.4	14.21	11.99
Telecommunications	12.65	12.76	12.43	12.58	12.65	19.26	19.49	18.79	14.34	16.5	14.61
IT and other information services	15.79	15.64	10.61	8.61	10.17	13.85	13.89	12.48	12.87	13.44	11.23

	IVA (% of gross exports)										
	2005	2006	2007	2008	2009	2010	2011	2012	2013	2014	2015
Czechia											
ICT services:	25.54	26.18	24.97	25.02	24.75	24.58	24.37	22.19	22.29	20.85	16.94
Telecommunications	15.73	15.25	14.28	15.31	15.19	14.5	15.51	15.99	15.47	16.06	14.4
IT and other information services	24.09	23.85	23.67	23.26	23.87	23.01	21.9	20.09	19.39	17.25	13.23
Estonia											
ICT services:	17.87	19.71	21	16.29	16.01	14.68	14.41	14.87	13.2	14.93	16.03
Telecommunications	13.95	16.53	19.39	14.19	14.38	12.03	12.19	12.66	12.37	15.22	17.34
IT and other information services	17.63	18.27	19.02	16.4	15.65	15.38	15.03	15.06	12.62	13.96	13.4
Hungary											
ICT services:	23.27	22.87	21.44	19.13	18.96	18	18.69	16.86	16	15.11	13.63
Telecommunications	18.72	20.61	20.72	18.62	19.4	20.15	19.76	19.89	20.57	18.4	13.94
IT and other information services	25.32	23.56	20.84	16.6	16.85	16.04	16.94	13.84	12.59	12.23	11.3
Latvia											
ICT services:	22.86	25.73	26.65	26.26	26.6	25.53	25.99	23.84	20.79	25.25	25.22
Telecommunications	18.69	20.61	24.3	23.35	25.17	25.21	26.6	27.96	25.3	33.33	33.77
IT and other information services	25.88	30.88	25.92	24.75	22.48	20.34	21.54	17.54	14.52	18.72	19.25
Lithuania											
ICT services:	17.62	18.81	19.8	20.61	20.83	19.17	20.38	20.26	23.25	24.9	21.22
Telecommunications	7.72	8.21	9.51	10.94	11.76	9.48	9.75	11.39	14.63	18.43	15.38
IT and other information services	30.15	29.97	30.33	26.84	28.77	25.51	26.07	25.37	25.99	24.94	20.3
Poland											
ICT services:	27.98	27.59	28.04	28.64	29.08	27.62	27.09	27.02	25.08	24.08	25.89
Telecommunications	24.5	24.47	28.32	29.19	28.94	33.44	36.24	37.54	38.05	34.95	31.07
IT and other information services	26.03	24.77	25.99	26.29	26.55	24.31	23.69	22.68	19.53	18.52	22.13
Slovakia											
ICT services:	23.78	20.17	18.05	20.2	20.11	22.97	24.22	19.83	20.81	20.73	22.05
Telecommunications	14.6	14.89	16.6	18.77	18.15	18.52	18.91	19.15	18.57	19.07	20.36
IT and other information services	22.97	19.47	15.75	17.14	16.99	20.78	21.69	17.67	17.28	18.69	21.09
Slovenia											
ICT services:	26.56	27	26.5	25.95	25.83	24.08	22.24	20.77	21.36	20.83	24.74
Telecommunications	22.73	23.67	23.35	22.14	22.94	21.22	19.08	18	19.07	17.98	24.58
IT and other information services	23.82	22.97	21.73	22.03	20.31	19.4	18.33	16.81	16.95	17.67	20.1
Bulgaria											
ICT services:	20.94	21.65	22.8	14.93	22.56	28.37	27.01	25.51	23.89	20.81	24.96
Telecommunications	19.3	19.02	18.33	11.73	19.09	26.17	23.58	25.38	26.19	20.09	22.24
IT and other information services	25.04	25.8	23.97	15	25.84	26.36	27.43	24.64	21.41	20.33	25.92
Croatia											
ICT services:	23.72	24.5	24.67	24.06	23.58	23.88	25.33	25.47	25.49	26.83	25.97
Telecommunications	18.93	21.31	22.62	19.07	18.5	19.42	22.64	23.46	24.34	26.02	27.17
IT and other information services	25.97	23.43	23.22	22.82	22.49	20.37	19.38	19.5	19.88	20.01	17.83
Romania											
ICT services:	14.76	15.01	13.68	14.32	15.87	26.95	26.66	26.41	26.22	28.53	20.64
Telecommunications	11.77	12.68	13.15	14.78	14.32	28.82	27.9	31.44	25.96	28.4	27.56
IT and other information services	18.11	18.5	12.78	13.01	16.76	25.22	24.68	23.89	26.26	28.41	18.41

	Relative position in GVCs										
	2005	2006	2007	2008	2009	2010	2011	2012	2013	2014	2015
Czechia											
ICT services:	0.07	0.06	0.06	0.07	0.06	0.04	0.04	0.02	0.02	0.01	− 0.02
Telecommunications	0.02	0.01	0.01	0.01	0.01	− 0.01	− 0.01	− 0.02	− 0.06	− 0.06	− 0.08
IT and other information services	0.07	0.06	0.05	0.06	0.06	0.03	0.03	0.02	0.01	0	− 0.02
Estonia											
ICT services:	− 0.04	− 0.01	0.02	− 0.01	− 0.01	− 0.05	− 0.07	− 0.05	− 0.07	− 0.06	− 0.04
Telecommunications	− 0.10	− 0.06	− 0.01	− 0.06	− 0.05	− 0.12	− 0.14	− 0.14	− 0.14	− 0.09	− 0.08
IT and other information services	− 0.03	0	0.03	0.03	0.03	0.02	0	0	− 0.03	− 0.05	− 0.04
Hungary											
ICT services:	0.07	0.06	0.04	0.01	0	0	0	− 0.02	− 0.02	− 0.03	− 0.06
Telecommunications	0.06	0.05	0.05	0.03	0.03	0.04	0.03	0.02	0.01	− 0.01	− 0.07
IT and other information services	0.08	0.06	0.03	− 0.01	− 0.01	− 0.01	0	− 0.02	− 0.03	− 0.02	− 0.07
Latvia											
ICT services:	0.1	0.1	0.11	0.12	0.12	0.1	0.11	0.08	0.06	0.11	0.11
Telecommunications	0.07	0.07	0.11	0.1	0.11	0.11	0.11	0.1	0.1	0.15	0.14
IT and other information services	0.13	0.13	0.1	0.13	0.12	0.07	0.08	0.05	0.02	0.09	0.08
Lithuania											
ICT services:	0.07	0.08	0.08	0.07	0.1	0.06	0.07	0.06	0.11	0.11	0.07
Telecommunications	− 0.02	− 0.02	− 0.01	0.02	0.03	− 0.01	− 0.02	0	0.05	0.06	0.02
IT and other information services	0.18	0.18	0.17	0.05	0.13	0.11	0.1	0.1	0.13	0.12	0.07
Poland											
ICT services:	0.13	0.11	0.12	0.12	0.13	0.1	0.1	0.1	0.09	0.08	0.09
Telecommunications	0.11	0.09	0.11	0.11	0.12	0.14	0.16	0.18	0.18	0.16	0.11
IT and other information services	0.11	0.09	0.1	0.11	0.11	0.08	0.07	0.07	0.06	0.04	0.07
Slovakia											
ICT services:	0.02	− 0.01	− 0.01	0.03	0.04	0.07	0.09	0.05	0.03	0.02	0
Telecommunications	− 0.01	− 0.04	− 0.03	0.03	0.04	0.07	0.08	0.06	0.04	0.03	− 0.01
IT and other information services	0.01	0	− 0.02	0	0.02	0.07	0.08	0.04	0	− 0.01	− 0.02
Slovenia											
ICT services:	0.06	0.06	0.05	0.06	0.05	0.03	0.01	− 0.01	− 0.01	− 0.01	0.02
Telecommunications	0.03	0.04	0.03	0.02	0.01	− 0.01	− 0.03	− 0.05	− 0.05	− 0.05	− 0.01
IT and other information services	0.07	0.07	0.05	0.06	0.04	0.03	0.02	0	0.01	0.02	0.03
Bulgaria											
ICT services:	0	0.02	0.03	0.01	0.06	0.12	0.13	0.09	0.1	0.05	0.06
Telecommunications	− 0.01	0	0	0.02	0.05	0.11	0.11	0.08	0.11	0.03	0.03
IT and other information services	0.02	0.05	0.05	− 0.01	0.07	0.13	0.15	0.1	0.09	0.06	0.08
Croatia											
ICT services:	0.06	0.08	0.08	0.08	0.09	0.1	0.1	0.1	0.1	0.11	0.1
Telecommunications	0.05	0.07	0.07	0.06	0.07	0.09	0.09	0.1	0.1	0.11	0.11
IT and other information services	0.09	0.07	0.08	0.08	0.08	0.09	0.07	0.07	0.07	0.07	0.05
Romania											
ICT services:	0.01	0.01	0.02	0.03	0.04	0.09	0.08	0.1	0.11	0.12	0.07
Telecommunications	− 0.01	0	0.01	0.02	0.01	0.08	0.07	0.1	0.1	0.1	0.11
IT and other information services	0.02	0.02	0.02	0.04	0.06	0.1	0.09	0.1	0.11	0.12	0.06

If the relative position in GVCs is higher than 0, it means that the country is located in the upstream market. If the relative position of GVCs is lower than 0, it means that the country is located in the downstream market. Source: Calculated by the author based on OECD’s Inter-Country Input–Output Database, 2020

When one analyses ICT services, the aspect of “servicification” of manufacturing is impossible to avoid. In 2005–2015, the CEE countries heavily utilized foreign ICT services in manufacturing. This phenomenon is visible in particular in the Visegrad countries, where roughly two-thirds of ICT services’ value added that flowed in form aboard became part of manufacturing, especially automotive and electronics. ICT services that were treated as a separate product were predominant in Estonia, Latvia, and Croatia. Moreover, in most CEE countries, dependence on foreign value added in ICT’s “servicification” of manufacturing has increased, which is reflected in the decline in the domestic value added of ICT services utilized in manufacturing. Only in Latvia and Romania, and slightly in Poland, ICT’s domestic value added prevailed—54.3%, 57.1%, and 50.6%, respectively. In turn, the most significant decrease in ICT services’ domestic value added was observed in Lithuania (22.2%). Romania (11.2%) and Bulgaria (6.4%) experienced the greatest improvement in this field, which implies that their manufacturing relied more and more on domestic ICT services (Table 3) (OECD, 2020).

**Table 3:** ICT “servicification” of manufacturing exports in the CEE economies in 2005–2015 (%)

	2005	2006	2007	2008	2009	2010	2011	2012	2013	2014	2015	Change between 2015 and 2005
Czechia	75.9	75.4	75.7	74.8	71.9	71.8	72.6	71.9	73.0	73.5	69.8	− 6.1
Estonia	41.9	44.8	46.4	46.0	44.6	48.7	53.8	51.7	51.5	49.8	45.5	3.6
Hungary	84.1	81.3	78.6	76.2	72.0	75.2	75.2	74.8	74.6	73.4	69.0	− 15.2
Latvia	41.9	39.3	42.5	39.0	32.1	34.7	37.0	36.3	36.0	38.1	33.7	− 8.2
Lithuania	61.3	57.6	53.9	60.0	56.6	59.8	62.0	60.7	64.1	57.9	56.9	− 4.5
Poland	67.6	67.8	67.7	67.7	65.1	63.7	64.0	64.1	64.5	61.1	60.8	− 6.8
Slovakia	78.7	81.2	81.1	78.2	81.8	83.4	86.5	81.8	80.1	81.6	77.9	− 0.7
Slovenia	70.0	71.3	70.4	66.6	62.7	62.6	62.3	58.9	58.9	57.7	56.6	− 13.5
Bulgaria	43.2	50.0	60.2	63.9	52.4	59.6	66.1	57.9	62.7	59.6	57.2	14.0
Croatia	36.5	34.8	37.3	36.2	33.8	44.2	36.2	33.2	35.6	34.5	34.5	− 2.0
Romania	70.5	66.9	64.1	47.2	45.0	52.8	55.7	61.2	58.2	54.8	47.1	− 23.3

As a percent of FVA. Source: Calculated by the author based on OECD’s Inter-Country Input–Output Database, 2020

This analysis examined if ICT services have become a new channel of improving CEE nations’ position in GVCs. The study shows that the hypothesis could not be true for all countries.

The V4 could have become some of the most specialized economies in software and ICT, R&D, or data centres in Europe. Four economies surpassed many developed countries in terms of ICT companies per 100 thousand population. Moreover, ICT expenditures as a percent of GDP in V4 were high (fDi Benchmark, 2020). In terms of an average connection speed (Ipv4, Mbps) and broadband adoption, the V4 exceed many EU-15 states (Akamai, 2016). Although the employment rate of the ICT sector remains the highest in north-western Europe, levels in the Visegrad countries are quickly catching up: Czechia (4.1%) is the top performer among the V4, ahead of Hungary (3.7%), Slovakia (3.2%), and Poland (3%) (Eurostat, 2020). In the Visegrad countries, there are local offices of many well-known international companies from the ICT industry, e.g., IBM, MSD, Samsung, HP, and Microsoft. These countries have also played the role of outsourcing centres (mainly IT support) for a large number of companies, e.g., Citibank, Getronic EMEA, Oracle, Tata Consulting, Unisys, ZTE, SAP, Siemens, Commerzbank, Phillips, Morgan Stanley, or KBC. Moreover, the V4 has established its own companies in ICT sectors, e.g., Avast Software, Seznam.cz, GoodData, Asseco, Poland, and Comarch. In this region, there operate special IT clusters or zones with preferential politics, e.g., North Hungarian IT Cluster, The Mazovia ICT Cluster or Wielkopolska ICT Cluster (Poland), Moravia Silesia Region or Prague (Czechia), and Žilina and Košice (Slovakia). Although the analysed states are important for international firms’ location, their position in the 2019 Global Service Location Index dropped (Kearney, 2019).

Despite the many advantages of the V4, it is difficult to perceive ICT services as an engine of growth and advancing their relative positions in GVCs. The V4’s focus on manufacturing, especially the automotive and electronics industries, has neglected the role of ICT services. Particularly unfavourable changes were observed in Czechia and Hungary, which experienced high increases in FVA in telecommunications (by 10.63% in Czechia and 10.66% in Hungary) and considerable decreases in IVA in IT and other information services (by 10.86% in Czechia and 14.02% in Hungary). It was connected with China and other developing countries moving up the value chain. Moreover, regional serious competitors with cost advantage have emerged in the field of ICT services outsourcing (e.g., Romania, the Baltic States). These factors caused the V4 to lose the relative position in the upstream market gradually. Only positive tendencies were observed in Poland and Slovakia, where a significant increase in IVA in telecommunications (by 6.57% in Poland and 5.76% in Slovakia) occurred, and in Czechia, where there was a slight decrease in FVA in IT and other information services (by 1.05%). Hence, the hypothesis that ICT services took over the role of a new channel, allowing for the improvement of the position of the V4 in GVCs, has not been confirmed, except for Poland. In fact, in the first years after accession to the EU, it was possible to suspect that ICT services’ role would have been important (Table 2), but the V4 has not introduced appropriate structural changes to economies to face foreign competition.

The Visegrad countries’ ICT services exports became quite dependent, like all of EU-11, on the EU’s value added, especially on German value added. Only in Slovakia did China surpass Germany as a prime supplier of value added to ICT services. It should not be surprising that Germany plays an important role in the Visegrad economies’ linkages to value chains. The geographic proximity, cultural similarities, and substantial labour cost differentials should be mentioned among the most important factors that led many German companies to shift large parts of their production to these countries and are responsible for closer economic integration between Germany and the Visegrad economies.

In 2015, Hungary relied on the EU’s value added the most (62.68%), and Czechia—the least (56.66%). The Visegrad countries did not cooperate with each other significantly in the field of ICT services, as evidenced by the low share of EU-11 value added in exports. Poland checked a particularly low level of added value from the EU-11 (6.14%). Apart from the EU value added, also the USA and China were the important suppliers of value added, especially for Czechia, Hungary, and Poland (Table 4).

**Table 4:** Top-3 suppliers of value added in total services and ICT services in 2015

	Czechia		Estonia		Hungary	
	ICT services	Total services	ICT services	Total services	ICT services	Total services
1	Germany (17.83%)	Germany (21.20%)	Finland (10.28%)	Finland (8.46%)	Germany (18.31%)	Germany (18.79%)
2	China (10.50%)	Russian Federation (8.54%)	Sweden (9.44%)	Germany (8.04%)	U.S. (8.21%)	Russian Federation (7.40%)
3	U.S. (8.92%)	China (6.80%)	U.S. (6.89%)	Sweden (7.32%)	China (6.43%)	U.S. (7.12%)
	EU-28 (56.66%)	EU-28 (60.17%)	EU-28 (60.31%)	EU-28 (59.44%)	EU-28 (62.68%)	EU-28 (64.47%)
	EU-11 (10.96%)	EU-11 (12.74%)	EU-11 (15.06%)	EU-11 (16.56%)	EU-11 (10.88%)	EU-11 (13.88%)
	Slovakia		Slovenia		Bulgaria	
	ICT services	Total services	ICT services	Total services	ICT services	Total services
1	China (11.56%)	Germany (12.98%)	Germany (10.04%)	Germany (12.52%)	Germany (10.88%)	Russian Federation (16.83%)
2	Germany (10.90%)	Czechia (10.07%)	Austria (7.64%)	Italy (9.74%)	Russian Federation (9.27%)	Germany (10.39%)
3	Czechia (8.45%)	Russian Federation (9.86%)	Italy (7.22%)	Austria (8.21%)	U.S. (6.03%)	Romania (5.25%)
	EU-28 (52.26%)	EU-28 (56.34%)	EU-28 (56.88%)	EU-28 (62.53%)	EU-28 (58.15%)	EU-28 (52.97%)
	EU-11 (16.82%)	EU-11 (19.94%)	EU-11 (14.73%)	EU-11 (14.44%)	EU-11 (12.86%)	EU-11 (12.70%)

	Latvia		Lithuania		Poland	
	ICT services	Total services	ICT services	Total services	ICT services	Total services
1	Russian Federation (11.31%)	Russian Federation (15.62%)	Russian Federation (11.75%)	Russian Federation (22.91%)	Germany (15.39%)	Germany (17.55%)
2	Estonia (8.34%)	Lithuania (9.19%)	Poland (7.79%)	Germany (6.29%)	China (8.90%)	Russian Federation (9.09%)
3	U.S. (8.07%)	Germany (6.78%)	U.S. (7.57%)	Poland (6.22%)	U.S. (7.24%)	China (7.41%)
	EU-28 (58.94%)	EU-28 (55.64%)	EU-28 (57.00%)	EU-28 (44.89%)	EU-28 (59.19%)	EU-28 (57.50%)
	EU-11 (23.56%)	EU-11 (23.59%)	EU-11 (22.08%)	EU-11 (15.81%)	EU-11 (6.14%)	EU-11 (7.50%)
	Croatia		Romania			
	ICT services	Total services	ICT services	Total services		
1	Germany (10.30%)	Germany (12.06%)	Germany (12.93%)	Germany (13.61%)		
2	Austria (9.18%)	Italy (8.06%)	France (9.59%)	France (9.48%)		
3	Slovenia (8.19%)	Austria (8.06%)	U.S. (7.09%)	Russian Federation (8.56%)		
	EU-28 (63.22%)	EU-28 (62.26%)	EU-28 (65.89%)	EU-28 (61.92%)		
	EU-11 (16.25%)	EU-11 (16.71%)	EU-11 (10.22%)	EU-11 (11.73%)		

Source: Calculated by the author based on OECD’s Inter-Country Input–Output Database, 2020

In 2015, the Visegrad countries supplied the EU mostly in value added. Czechia provided ICT services value added to Germany, Slovakia, and Poland. Hungary’s main partners were Germany, Netherlands, and Austria. In turn, Polish ICT services went to Germany, Switzerland, and Czechia. Slovakian services were directed to Germany, Czechia, and Hungary (OECD, 2020).

The Baltic States are perceived as some of the most economically stable outsourcing destinations. These countries are characterized by high levels of digital solution adoption and effective legislation that ensures data security and efficient business operations. Estonia is well known in the world as an e-country with a developed digital society stemming. In turn, Latvia is in the leading European economy for investment and revenue in the ICT industry, while Lithuania has the largest ICT industry in the Baltic States. This can be attributed to innovative practices and a stable, well-trained, and highly productive workforce. The ICT industry in Latvia has experienced significant growth in the past several years. ICT sector is expected to become a leading industry in the Baltic States. It is responsible for around 20% of their exports. The Baltic States’ ICT services have been dominated by services, computer programming, consulting, and telecommunications. In 2019 Global Service Location Index, Estonia placed the highest in EU-11 (12.), followed by Lithuania (16.) and Latvia (21.) (Kearney, 2019).

The Baltic States are known as the birthplace of Skype, TransferWise, Pipedrive, Cloutex, Click and Grow, Grabcad, Erply, Fortumo, Lingvist (Estonia), Ruptela UAB, Data House (Lithuania), Opus, Helmes and ADM (Latvia), etc. Moreover, the Baltic States became home for a wide array of foreign companies that opened their R&D centres there, e.g., Microsoft, SAP, Apple, HP, Lenovo, Microsoft, Sony Ericsson, Acronis, Parallels (Estonia), Google, AIG, Nasdaq, Uber, IBM, Wix, HP, Virtustream, Exadel and Unity (Lithuania), and IBM, PwC, Accenture, KPMG, Siemens, ABB (Latvia).

In the case of the Baltic States, there is a similar ambiguity as in the V4—Latvia and Lithuania used ICT services to improve their position in the GVCs, while Estonia did not. Latvia and Lithuania were constantly developing the ICT sector and trying to rely on FVA as little as possible. In the case of Estonia, although a well-developed country in terms of innovation, ICT services have not become an important export product to a much larger extent. Estonia used ICT services more for its internal needs rather than directing them abroad and integrating into the GVCs. Hence, it is difficult to perceive Estonia as the economy that confirms the hypothesis stated in this study. On the other hand, Latvia and Lithuania, despite the fluctuations in the relative position of ICT services in GVCs, can be considered as countries that confirm the research question (Table 2).

The Baltic States were also strongly connected with the EU markets in terms of ICT services exports, but these ties seemed to be weaker than in the case of the Visegrad states. The largest dependence could be observed in Estonia (60.31%). The Baltic States were also the only ones that did not have Germany among the most important suppliers of value added in ICT services; instead, they cooperated with neighbouring countries. Latvia and Lithuania cooperated mostly with the Russian Federation (11.31% and 11.75% of ICT services value added relied on Russia), while Estonia, with Finland (10.28%). The Baltic States (especially Lithuania and Latvia) cooperated significantly with each other in the field of ICT services, which proved the high share of EU-11 value added in exports (Table 4).

Generally, the Baltic States maintain close economic ties with neighbouring countries, but sometimes extend their cooperation to Asian economies, e.g., China or ASEAN. Estonia was a source of ICT services’ value added for Finland, Sweden, and Latvia. Latvia cooperated in ICT services with Estonia, Germany, and Lithuania. Lithuania, in turn, was characterized by two top partners from Europe: Germany and Estonia, and a partner from Asia—Singapore (OECD, 2020).

The last group turned out to be the most heterogeneous—Bulgaria, Croatia, Romania, and Slovenia. The fast development of ICT services in these countries is a result of systematic human development, well-developed ICT infrastructure, and government commitment to boosting this industry in these economies. The four countries are perceived as modest innovators in Europe with great prospects. Being the largest economy among these states, Romania has created ICT industry as a primary growth driver. In Romania, there can be found local offices of companies such as Oracle, Amazon, IBM, RBC, or Deutsche Bank, which have already leveraged the development potential of Romanian ICT specialists at a lower cost than in Western Europe, while maintaining high quality. They have also contributed to the initial development of the tech start-up environment. Generally, the concept of “Industry 4.0” is very popular in Romania (12,000 ICT companies, each year a thousand new companies start operating in this country; 202,000 ICT specialists) (Investormania, 2020). Similar to Romanian is the Bulgarian ICT sector, which is characterized as stable and constantly growing, making it one of the most profitable sectors in this country. Bulgaria has a long tradition in the ICT (as well as in electronics sectors) and is still known as the”Silicon Valley “ of South-Eastern Europe. Nowadays, Bulgaria is home to approximately 10,000 ICT companies, 70% of which are only exporting. A total of 70,000 people are employed in the ICT sector (Eurostat, 2020).

Slovenia and Croatia are characterized by smaller ICT industries. However, this sector is actually doing quite well and keeps growing in both countries. The Slovenian sector consists of about 24,000 employees and 3700 companies, while the Croatian sector employs 44,000 ICT specialists and more than 7400 companies (Investslovenia, 2020; Investcroatia, 2020). In Slovenia, Croatia, Bulgaria, and Romania, there are local offices of many well-known international companies from the ICT industry, e.g., Ericsson, HP, SAP, VMWare, Cisco, Atos, ProSys, Microsoft, or IBM.

The ways in which these countries joined ICT services’ GVCs were also different. Especially Romania is considered as the IT and outsourcing leader in CEE. Its comparative advantage is based on the highly skilled labour force, geographical and cultural proximity to Western Europe, and the welcoming business environment. Between 2005 and 2015, Slovenia became more depended on FVA. Generally, Slovenia reached the largest level of FVA in ICT services among all 11 economies (22.56% of gross exports). In Bulgaria, we saw an opposite tendency—country decreased its dependency on FVA in all ICT services subgroups. Other countries had mixed results depending on ICT services subgroup, e.g., Croatia decreased FVA in ICT services and increased FVA in telecommunications, while Romania increased FVA in telecommunications and decreased it in IT and other information services. A similar situation happened in IVA. In Slovenia, IVA dropped in all analysed industries, while in Bulgaria and Romania, it climbed significantly. Bulgaria, Croatia, and Romania rank relatively high in the GVCs. In Slovenia, the situation is ambiguous, because in regard to telecommunications, it is located in downstream market, but ICT services as a whole are positioned in upstream market and have recently proven a tendency to improve. It can be stated that all countries analysed in this indentation countries (with some reservation in relation to Slovenia) confirmed the second hypothesis (Table 2). Several factors contributed to the increase in the importance of ICT services as a channel of inclusion in the GVCs of the analysed countries, including the appropriate economic policy of these countries, establishing cooperation with countries outside of the EU (e.g., China, Russia), and structural changes.

All four economies were strongly connected to the EU markets in terms of ICT services exports, as much as the Visegrad countries. The largest dependence on European value added could be observed in Romania (65.89%). Moreover, Romanian ICT services were the most tied to European value added among all 11 countries. Like in the Visegrad states, Germany was also the leader among value-added suppliers to these four countries. They cooperate significantly with the other EU-11 countries in this field too, which proved the quite high share of value added in exports (Table 4).

Analysing the main recipients of value added from this group of countries, the EU also leads. Croatia cooperated with Slovenia, Austria, and Italy mostly, while Romanian ICT services were directed to Germany, France, and Italy. In turn, Slovenian and Bulgarian ICT services went to Germany, Austria, and Italy (OECD, 2020).

## Conclusions

The CEE countries have recently changed their role in the global economy. First, they were adversely affected by the financial crisis, although in the first years after the crisis, their GVC linkages became more advantageous. Second, they had to face strong Chinese and other low-cost producers’ competition, and in time, most of them increasingly relied on FVA. Then they needed to recover and modify their production patterns, because manufacturing GVCs became unfavourable for most of EU-11. This situation necessitated joining the global economy with a more sophisticated and advanced offer, in which services included ICT.

This study tries to verify two hypotheses: (1) the position of manufacturing in GVCs has been steadily weakening and (2) services, especially ICT services, can have a positive effect on participation of CEE economies in GVCs.

The first hypothesis was confirmed by most of countries. Unfortunately, the second hypothesis could not be true for all CEE countries, because some of them differed noticeably between each other with unfavourable connections in ICT services’ GVCs.

Several factors contributed to the increase in the importance of ICT services as a channel of inclusion in the GVCs of the analysed countries, including the appropriate economic policy, establishing cooperation with countries outside the EU (e.g., China, Russia), and structural changes. The ways in which these countries changed their economic structures to join ICT services’ GVCs were also different (Di Berardino & Onesti, 2020). In Poland, Latvia, Lithuania, Bulgaria, Croatia, Romania, and to some extend Slovenia (only telecommunications is in downstream market) ICT services and their subgroups have become an important positive force in their relative positions in GVCs (these countries are located in upstream market). Unfortunately, for the other four economies, connections within ICT services did not improve their relative position in GVCs.

Certainly, a number of restrictions of this study and areas for future research should be mentioned. First, the limited availability of statistical data, especially value-added flow statistics, was a serious obstacle. Second, the study relied on particular methods and it cannot be ruled out that applying different methodologies would lead to different conclusions. Third, this study should be repeated in the following years for confirmation results, especially after the COVID-19 crisis. Four, the study needs a more detailed examination of GVC interconnectivity between ICT services and other industries.

## Footnotes

1 According to the Swedish National Board of Trade (2016), under the term of “servicification” of manufacturing one understands that the manufacturing has been increasingly relying on service sector. In turn, Vandermerwe and Rada (1988) defined “servicification” of manufacturing as: “the increased offering of fuller market packages or ‘bundles’ of customer focused combinations of goods, services, support, self-service and knowledge in order to add value to core product offering.”.

2 The similarities and differences among the most popular value added databases presented (Jones et al., 2014).

3 For example, Czechia introduced the Strategy of Education Policy, Action Plan for the Development of Intelligent Transport Systems in the Czech Republic (ITS) by 2020 or Innovation Strategy of the Czech Republic 2019–2030. Industry 4.0 National Technology Platform Association or Digital Well-being Program was initiated in Hungary. Poland introduced the tool Direction Industry 4.0 or the Chamber of Commerce of Industry 4.0. Slovakia introduced, inter alia, Strategy of Digital Transformation of Slovakia, 2030.
